# Association Between Mitral Annular Calcification and Ventricular Tachycardia in Patients with Reduced and Mildly Reduced Ejection Fraction

**DOI:** 10.3390/jcm15031172

**Published:** 2026-02-02

**Authors:** Müjgan Ayşenur Şahin, Ahmet Seyda Yılmaz, Elif Ergül, Hakan Duman, Hüseyin Durak, Abuzer Duran, Şuayp Osmanoğlu, Mustafa Çetin

**Affiliations:** Department of Cardiology, Faculty of Medicine, Recep Tayyip Erdoğan University, Rize 53020, Türkiye; mujganaysenur.sahin@erdogan.edu.tr (M.A.Ş.); elif.ergul@erdogan.edu.tr (E.E.); hakan.duman@erdogan.edu.tr (H.D.); abuzer_duran23@erdogan.edu.tr (A.D.); suayp.osmanoglu@erdogan.edu.tr (Ş.O.);

**Keywords:** heart failure, mitral annular calcification, ventricular tachycardia, arrhythmia, inflammation

## Abstract

**Objective:** This study aimed to evaluate the association between mitral annular calcification (MAC) and ventricular tachycardia (VT) in patients with reduced and mildly reduced ejection fraction and to identify independent predictors of VT. **Materials and Methods**: A total of 143 patients with heart failure and left ventricular ejection fraction (LVEF) under 50% were included in this retrospective cross-sectional study. Patients were classified into two groups according to the presence of VT. Clinical, biochemical, and echocardiographic variables were compared between groups. Independent predictors of VT were identified using multivariable logistic regression analysis. **Results:** MAC was significantly more prevalent in the VT group compared with controls (43.6% vs. 17.4%, *p* < 0.001) and was the strongest independent predictor of VT (OR: 2.74; 95% CI: 1.13–6.65; *p* = 0.026). Higher inflammatory activity, lower serum albumin levels, increased left atrial volume, renal dysfunction, and elevated diastolic filling pressures were also associated with VT. **Conclusions**: MAC is a strong and independent predictor of ventricular tachycardia in patients with reduced and mildly reduced ejection fraction. Incorporating MAC into the overall arrhythmic risk profile alongside inflammatory, metabolic, and structural parameters may improve risk stratification in this population.

## 1. Introduction

Heart failure represents a heterogeneous clinical syndrome characterized by structural and functional myocardial impairment. Contemporary guidelines classify heart failure by left ventricular ejection fraction (LVEF) into reduced (HFrEF), mildly reduced (HFmEF), and preserved (HFpEF) categories; among these, patients with reduced or mildly reduced LVEF are known to exhibit more advanced ventricular remodeling, interstitial fibrosis, and electrical heterogeneity, all of which increase susceptibility to malignant ventricular arrhythmias such as ventricular tachycardia (VT) [1,2].

Ventricular tachycardia contributes substantially to sudden cardiac death in individuals with structural heart disease. Despite improvements in imaging, device therapy, and pharmacological management, accurately identifying patients at increased arrhythmic risk remains challenging. Easily accessible markers that reflect underlying structural and electrical instability are therefore of clinical importance [3,4].

Mitral annular calcification (MAC) is a chronic degenerative process characterized by calcium and fibrotic tissue deposition along the mitral annulus. Although traditionally viewed as a benign echocardiographic finding, accumulating evidence shows that MAC is associated with conduction abnormalities, atrial fibrillation, stroke, and other adverse cardiovascular outcomes [5]. Current international guidelines do not provide specific management recommendations for MAC as an isolated condition. Instead, MAC is considered a marker of advanced degenerative valvular disease and increased cardiovascular risk, and management strategies focus on optimal treatment of associated comorbidities and global cardiovascular risk reduction. Structural abnormalities of the mitral annulus, such as mitral annular disjunction and mitral valve prolapse, have also been linked to ventricular arrhythmias through mechanisms involving local fibrosis, mechanical stress, and conduction delay. Although MAC and mitral annular disjunction are distinct entities, both involve alterations of the mitral annulus that may contribute to arrhythmogenic remodeling. These observations raise the possibility that MAC may reflect a broader myocardial disease process extending beyond the annulus itself [6].

However, data on the relationship between MAC and ventricular tachycardia, particularly in patients with reduced or mildly reduced ejection fraction, remain limited and inconclusive. Because both MAC and VT share common pathways such as fibrosis, inflammation, and altered ventricular mechanics, investigating this potential relationship may offer clinically meaningful insights [7]. Therefore, the present study aimed to evaluate the association between the easily obtainable marker MAC and ventricular tachycardia and to identify independent predictors of VT in patients with reduced and mildly reduced ejection fraction.

## 2. Materials and Methods

### 2.1. Study Design

**Figure 1:**
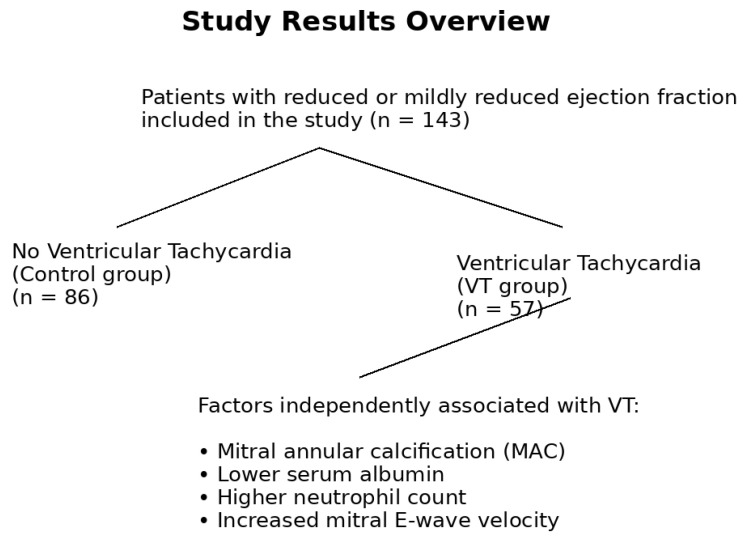
Overview of the study population, group classification, and variables independently associated with ventricular tachycardia.

This study was designed as a retrospective, observational, and cross-sectional investigation conducted between 1 February 2025 and 1 August 2025 at the Cardiology Department, Coronary Intensive Care Unit, and Cardiology Outpatient Clinic of Recep Tayyip Erdoğan University Training and Research Hospital. Consecutive patients with reduced or mildly reduced left ventricular ejection fraction who were followed at our institution and who were eligible for inclusion if they were ≥18 years of age, had available transthoracic echocardiographic data including assessment of mitral annular calcification, and had sufficient clinical follow-up for ventricular arrhythmia evaluation were included in the study. A total of 143 patients aged 18 years or older with LVEF below 50% were enrolled and classified into two groups based on the presence of ventricular tachycardia (VT) (Figure 1).

### 2.2. Diagnosis of Ventricular Tachycardia

The diagnosis of VT was confirmed through multiple diagnostic modalities. All VT episodes included in the study were monomorphic in morphology. Sustained VT was defined as a wide-complex tachycardia lasting at least 30 s, identified on rhythm strips obtained in the emergency department or intensive care unit. VT episodes recorded during 24–72 h Holter monitoring were also included. For patients with implanted pacemakers or implantable cardioverter-defibrillators (ICDs), intracardiac electrograms saved in device memory were reviewed by a device specialist, and ventricular arrhythmias were confirmed based on morphology and rate analysis.

### 2.3. Confirmation of Absence of VT in the Control Group

The absence of VT in the control group was verified through systematic rhythm assessment. All control patients underwent at least one 12-lead electrocardiogram showing no ventricular tachycardia or wide-complex tachycardia. For patients with cardiac devices, device interrogation demonstrated no recorded ventricular arrhythmias. None of the patients in the control group reported symptoms suggestive of VT during follow-up.

### 2.4. Clinical Definitions

Clinical diagnoses were established according to standardized international criteria. Hypertension was defined as a documented diagnosis, the use of antihypertensive medication, or repeated blood pressure measurements of 140/90 mmHg or higher. Diabetes mellitus was defined by a previously established diagnosis, ongoing antidiabetic therapy, fasting plasma glucose ≥ 126 mg/dL, or HbA1c ≥ 6.5% [8]. Dyslipidemia was accepted in the presence of a documented diagnosis, the use of lipid-lowering therapy, LDL-cholesterol ≥ 130 mg/dL, or triglyceride levels ≥ 150 mg/dL. Coronary artery disease was defined as angiographically confirmed stenosis of 50% or greater in at least one epicardial coronary artery, a history of myocardial infarction, or prior percutaneous coronary intervention (PCI) or coronary artery bypass grafting (CABG). Atrial fibrillation (AF) was defined as any AF episode documented on a 12-lead ECG, Holter monitoring, or device interrogation. Chronic kidney disease was defined according to KDIGO guidelines as an estimated glomerular filtration rate < 60 mL/min/1.73 m^2^ or previously confirmed CKD [9]. According to contemporary guideline definitions, heart failure is classified based on left ventricular ejection fraction (LVEF) into heart failure with reduced ejection fraction (HFrEF; LVEF < 40%), mildly reduced ejection fraction (HFmrEF; LVEF 40–49%), and preserved ejection fraction (HFpEF; LVEF ≥ 50%) [10]. Proteinuria was accepted in the presence of at least 1+ positivity on urine dipstick testing. Smoking status was categorized as current, former, or never smoker based on medical records, and at least one cigarette usage was considered to be a smoker. Prior stroke was defined as any confirmed ischemic or hemorrhagic cerebrovascular event. Chronic obstructive pulmonary disease was defined by a documented diagnosis or the use of long-term bronchodilator therapy.

### 2.5. Holter Monitoring

Patients presenting with symptoms suggestive of ventricular arrhythmias, such as palpitations, presyncope, syncope, or unexplained dizziness, underwent ambulatory electrocardiographic monitoring using a 24 to 72 h digital Holter system with a standard three-channel configuration. All Holter data were manually reviewed by an experienced cardiologist, artifacts were removed, and arrhythmic episodes were confirmed. Sustained VT was defined as a wide-complex tachycardia lasting at least 30 s or requiring urgent termination due to hemodynamic instability. Non-sustained VT was defined as three or more consecutive ventricular beats lasting less than 30 s. The Holter software (CardioScan Holter system (DM Software Inc., Stateline, NV, USA, http://www.dmsecg.com/products/holter/software.htm accessed on 10 January 2026)) automatically detected premature ventricular complexes (PVCs), couplets, triplets, and VT runs, and all automated detections were manually validated to ensure diagnostic accuracy. ICDs were implanted for both primary and secondary prevention according to guideline recommendations, and the ICD indication subgroups were not analyzed separately, as the study focused on VT occurrence rather than device indication.

### 2.6. Exclusion Criteria

Patients with arrhythmogenic cardiomyopathy and a history of chronic or acute pulmonary embolism, advanced hepatic failure, malignancy, active infection, collagen vascular diseases such as sarcoidosis and amyloidosis, chronic inflammatory or autoimmune disorders, endocrine abnormalities, or significant electrolyte disturbances (hypokalemia, hyperkalemia, hypocalcemia) were excluded. In addition, patients with recent stroke or transient ischemic attack, inadequate echocardiographic image quality, those who declined participation, and individuals younger than 18 years of age were not included in the study.

### 2.7. Laboratory Analysis

Venous blood samples were collected from all patients at the time of admission for routine biochemical analysis, complete blood count, cardiac biomarkers, and inflammatory markers. Hematological parameters were measured using an automated hematology analyzer based on impedance and flow cytometry principles for leukocyte differentiation and platelet counting. Serum biochemical parameters were analyzed using a fully automated biochemistry analyzer (Beckman Coulter AU5800, Brea, CA, USA) employing standard spectrophotometric and turbidimetric methods.

### 2.8. Transthoracic Echocardiography

All patients underwent comprehensive two-dimensional transthoracic echocardiography using a Philips EPIQ 7 ultrasound system (Philips Healthcare, Andover, MA, USA) equipped with a 2.5–3.5 MHz phased-array transducer. Standard parasternal long- and short-axis views and apical two-, three-, and four-chamber views were obtained in accordance with American Society of Echocardiography (ASE) guidelines. Measurements were performed during end-expiration with the patient in the left lateral decubitus position. Left atrial and left ventricular dimensions, as well as septal and posterior wall thicknesses, were obtained using M-mode echocardiography. Pulsed-wave Doppler and tissue Doppler imaging were used to assess diastolic parameters, including peak E and A velocities, E/A ratio, e′ velocities, and E/e′ ratio. LVEF was calculated using the biplane modified Simpson method. Left atrial volume was measured using the biplane area–length method from apical four- and two-chamber views at end-systole, and the left atrial volume index (LAVI) was obtained by normalizing this value to body surface area (mL/m^2^). Aortic valve sclerosis was defined as increased echogenicity and leaflet thickening without significant restriction of motion or hemodynamic stenosis. MAC was defined as bright, dense echogenic structures with acoustic shadowing localized along the posterior mitral annulus and extending into the mitral leaflet base when present [10].

### 2.9. Data Collection

Data were prospectively collected between 1 February 2025 and 1 August 2025 from clinical evaluations performed in the Cardiology Service, Coronary Intensive Care Unit, and Cardiology outpatient clinic. Electrocardiographic recordings, transthoracic echocardiographic images, and laboratory parameters, including hemogram, electrolytes, and renal function tests, were systematically reviewed for all participants. Patient information was recorded using standardized data collection forms and included clinical diagnosis at presentation, comorbidities, medical therapy, LVEF, presence or absence of VT, and echocardiographic findings indicating MAC. All data were anonymized and securely stored before transfer to statistical software for analysis.

### 2.10. Statistical Analysis

All statistical analyses were conducted using SPSS version 25.0 (IBM Corp., Armonk, NY, USA). The Kolmogorov–Smirnov test was applied to assess the distribution characteristics of continuous variables. Normally distributed variables were expressed as mean ± standard deviation, whereas non-normally distributed variables were presented as median (minimum–maximum). Patients were categorized into two groups based on the presence or absence of VT. Categorical variables were compared using the Pearson chi-square test or Fisher’s exact test when appropriate. Continuous variables were compared using the independent samples *t*-test for normally distributed variables and the Mann–Whitney U test for non-normally distributed variables. Variables including renal function markers (creatinine, eGFR), inflammatory indices (white blood cell count, neutrophils, monocytes), metabolic markers (albumin, T3, T4), and echocardiographic parameters (LVEF, left atrial volumes, E/e′ ratio, LAVI, and presence of MAC) were analyzed. Independent predictors of VT were identified using a two-step logistic regression model. First, univariate analysis was performed; parameters including age, NYHA functional class, statin use, renal function parameters, inflammatory markers, echocardiographic indices, and mitral annular calcification were analyzed, and variables with *p* < 0.10 were included in the multivariable analysis. In the second step, MAC, LVEF ≤ 35%, proteinuria, LAVI, E-wave velocity, E/e′ ratio, albumin, creatinine, neutrophils, monocytes, age, and sex were entered into a backward stepwise multivariable logistic regression analysis. Odds ratios (OR) with 95% confidence intervals (CI) were reported, and a two-tailed *p* < 0.05 was considered statistically significant. In addition, receiver operating characteristic (ROC) curve analysis was performed only for variables that remained independently associated with ventricular tachycardia in the final multivariable logistic regression model. This approach was chosen to ensure consistency between regression and discrimination analyses and to avoid overfitting, given the limited number of events.

## 3. Results

### 3.1. Demographic and Clinical Characteristics

A total of 143 patients diagnosed with heart failure were included in the study. Among them, 57 patients (39.9%) had ventricular tachycardia, while 86 patients (60.1%) formed the control group without VT. The mean age of the VT group was 67.7 ± 11.8 years, and the control group had a mean age of 64.2 ± 10.2 years, a difference that was not statistically significant (*p* = 0.066). There were also no significant differences between the groups with respect to sex distribution (80.2% vs. 86% male percentage, *p* = 0.357). Body mass index (*p* = 0.900) and waist circumference (*p* = 0.146) were also similar between groups (Table 1).

Cardiovascular risk factors, including hypertension (65.1% vs. 71.9%, *p* = 0.393), diabetes mellitus (36% vs. 35.1%, *p* = 0.526), smoking (50% vs. 54.4%, *p* = 0.366), and dyslipidemia (53.5% vs. 56.1%, *p* = 0.445), showed similar distribution between the VT and control groups. Although non-ischemic cardiomyopathy (11.6% vs. 21.1%, *p* = 0.099) and atrial fibrillation (26.7% vs. 36.8%, *p* = 0.137) were detected to be more frequent in the VT group, these differences did not reach statistical significance. Functional capacity assessed by NYHA classification demonstrated a higher proportion of class III–IV patients in the VT group (21.1% vs. 9.3%, *p* = 0.042). Ischemic heart disease-related variables, including history of myocardial infarction (62.8% vs. 57.9%, *p* = 0.339), prior PCI (57% vs. 42.1%, *p* = 0.058), history of CABG (18.6% vs. 28.1%, *p* = 0.131), and angiographically confirmed coronary artery disease (68.6% vs. 78.9%, *p* = 0.176), showed no significant differences between the groups. The most striking clinical difference was the markedly higher rate of ICD implantation in the VT group (54.4% vs. 4.7%, *p* < 0.001). Regarding medication use, statin therapy was significantly more common in the control group (67.4% vs. 47.4%, *p* = 0.013). Use of other cardiovascular medications, including beta-blockers, angiotensin-converting enzyme inhibitors/angiotensin receptor blockers, thiazide diuretics, and nitrates, did not differ significantly between groups (*p* > 0.05 for each medication) (Table 1).

### 3.2. Biochemical and Hematological Parameters

Patients with VT indicated significantly worse renal function and higher inflammatory status. Serum creatinine levels were higher in the VT group (1.4 ± 0.71 vs. 1.13 ± 0.36 mg/dL, *p* = 0.003), while estimated glomerular filtration rate was lower (60 ± 25.8 vs. 70 ± 21.1 mL/min/1.73 m^2^, *p* = 0.013). Serum albumin levels were significantly reduced in the VT group (3.89 ± 0.54 vs. 4.68 ± 0.44 g/dL, *p* = 0.002). Inflammatory markers, including white blood cell count (9.2 ± 4.7 vs. 7.69 ± 1.9 × 10^3^/µL, *p* = 0.006), neutrophil count (5.9 ± 2.4 vs. 4.9 ± 1.5 × 10^3^/µL, *p* = 0.004), and monocyte count (0.59 ± 0.22 vs. 0.52 ± 0.15 × 10^3^/µL, *p* = 0.031), were significantly elevated in the VT group. Proteinuria was more frequent in the VT group compared to controls (41.1% vs. 20.2%, *p* = 0.007). Thyroid function analysis revealed significantly lower T3 levels (2.85 ± 0.62 vs. 3.10 ± 0.45 ng/dL, *p* = 0.002) and higher T4 levels (1.36 ± 0.30 vs. 1.20 ± 0.21 ng/dL, *p* = 0.003) in the VT group, while TSH values (1.18 (0.76–2.16) vs. 1.5 (0.68–2.09), *p* = 0.119) did not differ significantly. Other biochemical markers, including glucose, uric acid, hemoglobin, and lymphocyte count, showed no significant between-group differences (*p* > 0.05 for all) (Table 2).

### 3.3. Echocardiographic Findings

Echocardiographic parameters revealed lower mean LVEF in the VT group (35.4 ± 9.2 vs. 38 ± 7.8%), although this difference did not reach statistical significance (*p* = 0.074). However, the ratio of patients with LVEF ≤ 35% (31.4% vs. 48.2%, *p* = 0.033) and left atrial volumes, including maximum (24.7 ± 7.4 vs. 29.1 ± 9.2 mL, *p* = 0.003), minimum (19.2 ± 6.1 vs. 22.9 ± 8.1 mL, *p* = 0.003), and pre-A volumes (22.2 ± 6.5 vs. 26.1 ± 8.5 mL, *p* = 0.004), was significantly higher in the VT group. In addition, mitral E-wave velocity (76.2 ± 27.6 vs. 95.9 ± 37.5 cm/s, *p* < 0.001) and E/e′ ratio (10.5 ± 4.9 vs. 13.07 ± 7.22, *p* = 0.013) were also elevated in the VT group. There were no significant differences in the prevalence of moderate or severe valvular heart disease (45.3% vs. 47.4%, *p* = 0.463) or aortic valve sclerosis (37.2% vs. 52.7%, *p* = 0.051) between groups (Table 3).

### 3.4. Association Between MAC and Ventricular Tachycardia

**Figure 2:**
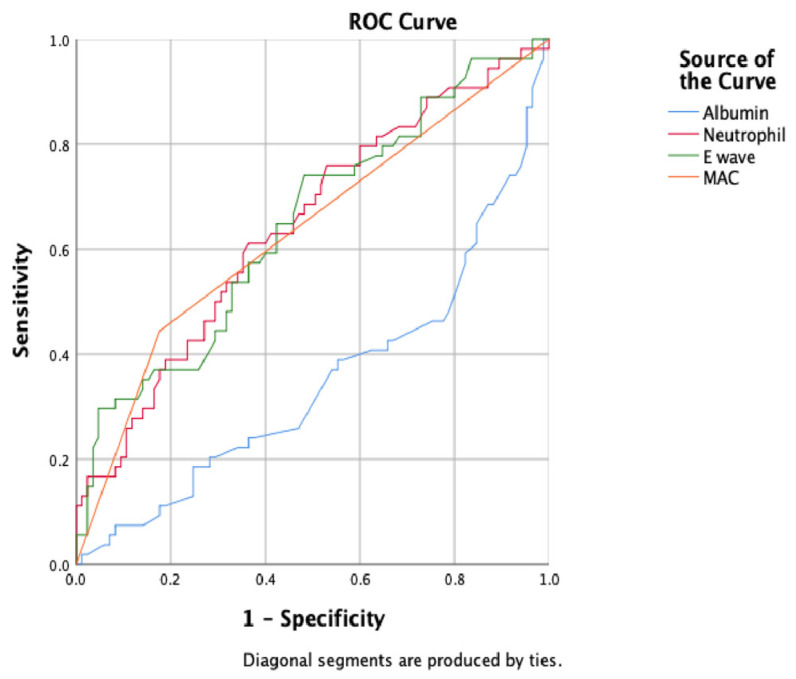
Receiver operating characteristic (ROC) curves for albumin, neutrophil count, mitral inflow E-wave velocity, and mitral annular calcification (MAC) in predicting ventricular tachycardia.

The prevalence of MAC was significantly higher in patients with VT compared with controls (15 (17.4%) vs. 24 (43.6%), *p* < 0.001). Multivariable logistic regression analysis demonstrated that MAC was an independent predictor of VT (odds ratio (OR) = 3.665, 95% confidence interval (CI):1.695–7.921, *p* < 0.001). ROC curve analysis was conducted for variables that remained significant in the final multivariable logistic regression model, including mitral annular calcification, serum albumin level, neutrophil count, and mitral E-wave velocity, and it was revealed that MAC had a modest but significant predictive performance for VT, with an area under the curve (AUC) of 0.634 (95% CI:0.537–0.731, *p* = 0.008) (Table 4), (Figure 2).

### 3.5. Independent Predictors of VT

Variables that demonstrated a *p*-value < 0.10 in the univariate analysis were included in the multivariable logistic regression model. The following factors independently predicted the presence of ventricular tachycardia: MAC (OR: 2.742; 95% CI: 1.130–6.653; *p* = 0.026), mitral E-wave velocity (OR: 1.019; 95% CI: 1.005–1.033; *p* = 0.009), neutrophil count (OR: 1.279; 95% CI: 1.029–1.589; *p* = 0.027), and serum albumin (OR: 0.372; 95% CI: 0.163–0.850; *p* = 0.019) (Table 4) (Figure 1).

## 4. Discussion

In this study, we demonstrated that the presence of MAC is independently associated with the development of VT in patients with reduced and mildly reduced ejection fraction. Additionally, lower serum albumin levels, higher neutrophil counts, and elevated mitral E-wave velocity were identified as independent predictors of VT. These results suggest that both the localized fibrotic-calcific substrate created by MAC and the concomitant systemic inflammation may contribute to arrhythmogenesis in the ventricle.

Heart failure is a progressive clinical syndrome that unsettles the structural and functional integrity of the cardiovascular system, initiating multidimensional hemodynamic, neurohormonal, and cellular adaptation processes [11,12]. VT occurs more frequently in patients with reduced ejection fraction due to structural changes such as increased ventricular wall stress, myocyte hypertrophy, fibrosis, and electrical heterogeneity. These alterations weaken the ventricular pump function and promote the formation of an arrhythmogenic substrate. Changes in conduction properties within the remodeled ventricular tissue, autonomic imbalance, and activation of inflammatory pathways play critical roles in the development of malignant arrhythmias such as VT [4,13]. Therefore, echocardiographic, laboratory, and clinical markers that reflect various aspects of cardiac remodeling are valuable in predicting arrhythmic risk in patients with reduced or mildly reduced ejection fraction.

Although MAC was once regarded as a benign degenerative process, accumulating evidence now indicates a strong association with adverse cardiovascular outcomes [14]. MAC has also been linked to impaired left ventricular function, particularly in patients with comorbid conditions such as diabetes, hypertension, and metabolic syndrome, where it is associated with reduced ejection fraction and poorer clinical prognosis. Proposed mechanisms include enhanced myocardial fibrosis, slowed electrical conduction, and abnormalities in ventricular repolarization, all of which may contribute to structural and electrophysiological deterioration [15,16,17]. A meta-analysis by Wang et al. demonstrated that MAC is significantly associated with all-cause and cardiovascular mortality, myocardial infarction, atrial fibrillation, and heart failure [18]. Similarly, the Northern Manhattan Study (NOMAS) showed that MAC is associated with increased risk of myocardial infarction and vascular mortality [19]. These data support the view that MAC is not only a structural cardiac finding but also a potential contributor to electrical instability and ventricular arrhythmias. Nonetheless, in the absence of direct myocardial substrate assessment, these observations should be interpreted cautiously, and MAC may primarily represent a marker of advanced myocardial disease and structural remodeling rather than a direct arrhythmogenic structure.

Although few studies have directly examined the relationship between MAC and VT, research on arrhythmic mitral valve prolapse and mitral annular disjunction has demonstrated that structural abnormalities of the mitral annulus can promote ventricular arrhythmias through low-voltage scarring, mechanical stretch, and conduction delay [20,21]. Previous studies have primarily focused on patients with preserved ejection fraction and structurally different substrates. These studies have demonstrated associations between ventricular arrhythmias and localized myocardial fibrosis or mechanical stretch near the mitral annulus. In contrast, our study specifically evaluated mitral annular calcification in a heart failure population with reduced or mildly reduced ejection fraction, reflecting a more advanced stage of myocardial disease. Unlike prior investigations that emphasized primary mitral valve pathology, our findings suggest that MAC may represent a marker of global structural remodeling and arrhythmic vulnerability rather than a localized arrhythmogenic trigger [22]. These differences in patient selection, disease stage, and study design may partly explain variations in reported arrhythmic associations across studies. These observations suggest that the mitral annulus may serve as an arrhythmogenic region, and MAC could represent another anatomical variant contributing to this substrate. The contribution of MAC to VT development can be explained by multifactorial mechanisms. Calcification may disrupt electrical conduction around the mitral annulus, enabling heterogeneous excitability and creating potential reentry circuits. MAC may also affect atrioventricular conduction pathways, contribute to local conduction slowing, and enhance electrical heterogeneity. As calcification progresses, fibrotic tissue accumulation around the annulus may also increase left ventricular wall stress and contribute to ventricular remodeling. This remodeling may result in myofiber disorganization, interstitial fibrosis, and conduction heterogeneity. Additionally, due to the anatomical proximity of the posterior and inferobasal left ventricular segments to the calcified mitral annulus, localized repolarization dispersion and transmural electrical instability may occur in these regions. Histopathological studies have demonstrated pronounced fibrosis, adipose infiltration, and localized scar tissue in myocardial regions associated with MAC [23,24,25]. Our findings indicate that the presence of MAC is independently associated with VT in patients with reduced ejection fraction. This association suggests that MAC may serve as a marker of increased arrhythmic vulnerability, and it can be regarded as a complementary marker reflecting structural disease burden rather than a standalone discriminator for clinical decision-making, including the need for closer rhythm monitoring and more careful consideration of ICD eligibility in this population.

Additionally, lower albumin levels and higher white blood cell, neutrophil, and monocyte counts in the VT group suggest that inflammation is an important factor contributing to arrhythmogenesis. Albumin is not only a marker of nutritional and hepatic status but also a potent antioxidant, anti-inflammatory, and endothelial-protective molecule. Low albumin levels have been shown to be associated with increased oxidative stress, endothelial dysfunction, impaired microvascular perfusion, and myocardial fibrosis. It was also indicated that hypoalbuminemia increases mortality and arrhythmic risk in heart failure [26,27]. The association between lower albumin levels and VT in our study aligns with these findings. Thus, it can be speculated that lower albumin levels may increase mortality rates in these patient groups by stimulating ventricular arrhythmic events. Furthermore, elevated inflammatory markers, including neutrophils and monocytes, further support the role of inflammation in VT development. Inflammation contributes to myocardial fibrosis, electrical heterogeneity, and conduction slowing, thereby facilitating arrhythmogenesis [28]. Increased neutrophil counts have been found to be associated with VT and sudden cardiac death, both in acute coronary syndromes and chronic heart failure. Therefore, the relationship between elevated inflammatory markers and VT in our study supports the central role of inflammation in both substrate formation and arrhythmic triggers.

Furthermore, the association observed between NYHA functional class and the presence of VT suggests that increasing symptom severity in heart failure may have a significant impact on arrhythmogenic vulnerability. In our study, patients with advanced symptoms (NYHA class III–IV) were more frequently represented in the VT group, supporting the notion that neurohormonal activation, ventricular remodeling, and electrical instability become more prominent in the later stages of the disease. Increased volume overload, greater fibrotic tissue accumulation, elevated intracardiac pressures, and autonomic imbalance are known to facilitate both the initiation of arrhythmias and the activation of existing arrhythmogenic substrates [29]. Therefore, this finding indicates that VT development is closely related not only to structural or biochemical markers but also to the clinical severity of heart failure symptoms.

When medication profiles were examined, statin therapy was significantly more common in the control group than in the VT group. However, the use of other cardiovascular and metabolic medications such as beta-blockers, ACE inhibitors/ARBs, thiazide diuretics, nitrates, and antidiabetic agents did not differ significantly between the groups. The similar distribution of clinically relevant therapies such as antidiabetic, anti-ischemic, and antiarrhythmic medications suggests that overall treatment strategies were largely comparable. This finding also indicates that current therapeutic approaches may not sufficiently prevent VT development and that deeper pathophysiological mechanisms such as structural cardiac remodeling, inflammation, and electrical instability—processes that may not be fully controlled by pharmacological therapy—continue to drive arrhythmogenesis. Although the higher prevalence of statin use in the control group may reflect closer monitoring of atherosclerotic risk, this difference did not transform into a significant impact on VT incidence. This suggests that mechanisms underlying VT extend beyond atherosclerotic burden, emphasizing the need for more specific and larger-scale studies to better understand the relationship between medication therapy and arrhythmic risk, particularly across different patient subgroups and drug classes.

The interplay between heart failure and ventricular arrhythmias is also strongly influenced by cardiorenal interactions. In our study, patients with VT had significantly higher serum creatinine levels, markedly lower glomerular filtration rates, and increased proteinuria, suggesting reduced renal reserve and its potential contribution to arrhythmogenic processes. Renal dysfunction promotes arrhythmia not only through uremic toxin accumulation and electrolyte disturbances but also by enhancing inflammation, oxidative stress, endothelial dysfunction, and myocardial fibrosis, which accelerate ventricular remodeling and increase susceptibility to arrhythmias [30]. Correspondingly, the significantly higher prevalence of proteinuria in the VT group is noteworthy, as proteinuria reflects both glomerular injury and systemic inflammation with vascular dysfunction. The higher inflammatory burden and impaired renal function observed in the VT group suggest that this metabolic stress profile may play an active role in promoting VT. Therefore, assessing renal function is essential for arrhythmic risk stratification, especially in patients with reduced or mildly reduced ejection fraction.

An increase in left atrial volume in the VT group reflects elevated atrial pressure and chronic diastolic dysfunction. Left atrial enlargement is not only a marker of diastolic impairment but also an indicator of atrial wall stress, fibrotic remodeling, and electrical heterogeneity. These structural changes are well known to increase susceptibility to atrial arrhythmias, and in the setting of advanced structural heart disease, such atrial electrical and mechanical abnormalities may further contribute to a myocardial environment that facilitates ventricular arrhythmogenesis through shared remodeling pathways [31]. The elevated E/e′ ratio in the VT group further supports the presence of increased ventricular filling pressures and a hemodynamic milieu conducive to electrical instability. Although left atrial enlargement was not an independent predictor of VT in our analysis, it likely represents a structural component within the broader arrhythmogenic substrate observed in patients with reduced ejection fraction.

In our study, ischemic heart disease variables, including prior myocardial infarction, PCI, or CABG, did not differ significantly between groups. This suggests that as heart failure progresses, structural remodeling, inflammation, and electrical heterogeneity may have a more prominent role in VT development than ischemic etiology alone, regardless of the underlying cause. On the other hand, the significantly higher rate of ICD implantation in the VT group reflects a clinical approach primarily oriented toward secondary prevention. More than half of the VT group had received ICD implantation, indicating that these patients had previously experienced life-threatening arrhythmias. In contrast, the very low rate of ICD implantation in the control group suggests firmer patient selection for primary prevention. Although primary prevention ICDs reduce mortality in selected heart failure patients, factors such as cardiomyopathy type, comorbidities, life expectancy, and cost-effectiveness influence decisions. The identification of high-risk patients earlier in the disease course, such as those with MAC and reduced EF, may prompt reconsideration of primary prevention strategies.

In addition, thyroid function parameters showed that T3 levels were lower while T4 levels were higher in the VT group, whereas TSH levels did not differ significantly. This pattern is consistent with the low T3 syndrome, also known as the euthyroid sick syndrome. Previous studies have shown that low T3 levels in heart failure are associated with increased inflammation, oxidative stress, impaired cardiac function, and worse clinical outcomes [32]. This finding highlights the possibility that VT development may not only be linked to structural and electrical mechanisms but also to metabolic and hormonal dysregulation.

From a research and clinical perspective, future studies should explore the potential role of mitral annular calcification in cardiovascular risk assessment related to sport participation and exercise prescription. Current recommendations for athletes with valvular heart disease emphasize individualized evaluation based on valve morphology, ventricular function, symptoms, and arrhythmic burden. In this context, MAC may represent an additional structural marker reflecting underlying myocardial vulnerability rather than isolated valvular degeneration. Prospective studies incorporating exercise testing, rhythm monitoring, and advanced imaging may help clarify whether MAC contributes incremental information to arrhythmic risk stratification during pre-participation assessment, particularly in individuals engaged in competitive or high-intensity sports. Such investigations could ultimately inform tailored exercise recommendations and refine risk-based decision-making frameworks, as highlighted in recent reviews on sport participation in mitral valve disease [33].

## 5. Limitations

This study has several limitations. First, the single-center design restricts the generalizability of the findings. Larger, multicenter studies with broader sample sizes are needed to validate the results. In addition, the assessment of MAC was performed solely using transthoracic echocardiography. The use of advanced imaging modalities such as computed tomography or cardiac magnetic resonance imaging could have provided a more objective and detailed evaluation of the extent and depth of calcification. Moreover, because electrophysiological studies were not performed, precise localization and mechanisms of VT could not be identified. A significant portion of the study population consisted of elderly individuals, which inherently increases the likelihood of both MAC and VT, raising the possibility that the observed association may be influenced by shared risk factors such as age. Another limitation is that patients with normal ejection fraction were not included. These individuals typically have lower arrhythmic risk and are followed less frequently, which increases the possibility that VT episodes may go undetected. Therefore, sufficient data for this group could not be collected. In addition, chronic amiodarone therapy was almost exclusively used in patients with documented ventricular tachycardia, whereas no patients in the control group received amiodarone. Accordingly, antiarrhythmic drug use represents post-diagnostic treatment rather than a baseline characteristic, and statistical comparison or adjustment was not considered methodologically appropriate due to the risk of treatment indication bias.

## 6. Conclusions

This study demonstrated that the presence of MAC is significantly associated with ventricular tachycardia in patients with reduced and mildly reduced ejection fraction. MAC may serve as an easily identifiable echocardiographic marker of arrhythmic vulnerability in this population. Patients with concomitant MAC and low ejection fraction may therefore benefit from closer rhythm surveillance, including more frequent ECG and Holter monitoring. In addition, the VT group exhibited higher left atrial volumes, impaired renal function, and elevated inflammatory markers. These observations indicate that, beyond structural abnormalities, inflammatory status and reduced renal capacity may further contribute to arrhythmic susceptibility in patients with reduced ejection fraction.

## Figures and Tables

**Table 1:** Demographic and Clinical Characteristics of Heart Failure Patients with and without Ventricular Tachycardia.

Variable	VT (−) (n = 86)	VT (+) (n = 57)	*p*-Value
Male sex, n (%)	69 (80.2)	49 (86.0)	0.357
Age (years)	64.2 ± 10.2	67.7 ± 11.8	0.066
BMI (kg/m^2^)	29.2 ± 4.9	29.3 ± 4.8	0.900
Waist circumference (cm)	104.8 ± 10.9	107.7 ± 12.8	0.146
Hypertension, n (%)	56 (65.1)	41 (71.9)	0.393
Diabetes mellitus, n (%)	31 (36.0)	20 (35.1)	0.526
Smoking, n (%)	43 (50.0)	31 (54.4)	0.366
Dyslipidemia, n (%)	46 (53.5)	32 (56.1)	0.445
History of atrial fibrillation, n (%)	23 (26.7)	21 (36.8)	0.137
Non-ischemic cardiomyopathy, n (%)	10 (11.6)	12 (21.1)	0.099
History of PCI, n (%)	49 (57.0)	24 (42.1)	0.058
History of MI, n (%)	54 (62.8)	33 (57.9)	0.339
History of CABG, n (%)	16 (18.6)	16 (28.1)	0.131
Coronary artery disease, n (%)	59 (68.6)	45 (78.9)	0.176
NYHA Class I, n (%)	39 (45.3)	24 (42.1)	0.417
NYHA Class II, n (%)	39 (45.3)	21 (36.8)	0.202
NYHA Class III–IV, n (%)	8 (9.3)	12 (21.1)	0.042
PAD, n (%)	3 (3.5)	4 (7.0)	0.283
Alcohol use, n (%)	6 (7.0)	5 (8.8)	0.463
History of stroke, n (%)	7 (8.1)	8 (14.0)	0.197
COPD, n (%)	7 (8.1)	4 (7.0)	0.537
ICD, n (%)	4 (4.7)	31 (54.4)	<0.001
Aspirin, n (%)	53 (61.6)	29 (50.9)	0.136
P2Y12 inhibitors, n (%)	29 (33.7)	13 (22.8)	0.111
Beta-blockers, n (%)	70 (81.4)	43 (75.4)	0.258
Calcium channel blockers, n (%)	18 (20.9)	12 (21.1)	0.573
ACE inhibitors, n (%)	35 (40.7)	22 (38.6)	0.470
ARBs, n (%)	23 (26.7)	10 (17.5)	0.141
Statins, n (%)	58 (67.4)	27 (47.4)	0.013
Thiazide diuretics, n (%)	17 (19.8)	11 (19.3)	0.561
Nitrates, n (%)	4 (4.7)	4 (7.0)	0.401
Warfarin, n (%)	5 (5.8)	7 (12.3)	0.145
DOACs, n (%)	12 (14.0)	7 (12.3)	0.490
Spironolactone, n (%)	23 (26.7)	14 (24.6)	0.464
Empagliflozin, n (%)	3 (3.5)	6 (10.5)	0.091
Dapagliflozin, n (%)	8 (9.3)	9 (15.8)	0.181
Other OADs, n (%)	9 (10.5)	7 (12.3)	0.468
Insulin, n (%)	5 (5.8)	4 (7.0)	0.516
Trimetazidine, n (%)	3 (3.5)	4 (7.0)	0.283
Previous VT, n (%)	0	23 (40.4)	<0.001
Furosemide, n (%)	23 (26.7)	17 (29.8)	0.414

BMI: Body mass index; PCI: Percutaneous coronary intervention; MI: Myocardial infarction; CABG: Coronary artery bypass grafting; NYHA: New York Heart Association; PAD: Peripheral artery disease; COPD: Chronic obstructive pulmonary disease; ICD: Implantable cardioverter-defibrillator; ACE: Angiotensin-converting enzyme; ARB: Angiotensin receptor blocker; DOAC: Direct oral anticoagulant; OAD: Oral antidiabetic drug; VT: Ventricular tachycardia.

**Table 2:** Laboratory findings of heart failure patients with and without ventricular tachycardia.

Variable	VT (−) (n = 86)	VT (+) (n = 57)	*p*-Value
Glucose (mg/dL)	134.6 ± 71.1	132.7 ± 56.3	0.864
Serum creatinine (mg/dL)	1.13 ± 0.36	1.41 ± 0.71	0.003
eGFR (mL/min/1.73 m^2^)	70 ± 21.1	60 ± 25.8	0.013
Total bilirubin (mg/dL)	0.96 ± 0.89	0.76 ± 0.37	0.128
Indirect bilirubin (mg/dL)	0.68 ± 0.55	0.57 ± 0.30	0.173
Albumin (g/dL)	4.68 ± 0.44	3.89 ± 0.54	0.002
Uric acid (mg/dL)	6.5 ± 2.1	6.7 ± 2.4	0.496
Proteinuria, n (%)	17 (20.2)	23 (41.1)	0.007
WBC (10^3^/µL)	7.69 ± 1.9	9.2 ± 4.7	0.006
Neutrophils (10^3^/µL)	4.9 ± 1.5	5.9 ± 2.4	0.004
Lymphocytes (10^3^/µL)	2.1 ± 1.6	2.07 ± 1.1	0.893
Hemoglobin (g/dL)	13.7 ± 1.8	13.1 ± 2.2	0.110
Monocytes (10^3^/µL)	0.52 ± 0.15	0.59 ± 0.22	0.031
TSH (mIU/mL)	1.18 (0.76–2.16)	1.5 (0.68–2.09)	0.119
T3 (ng/dL)	3.1 ± 0.45	2.85 ± 0.62	0.002
T4 (ng/dL)	1.2 ± 0.21	1.36 ± 0.30	0.003
HbA1c (%)	7.08 ± 1.7	6.8 ± 1.4	0.467

eGFR: Estimated glomerular filtration rate; WBC: White blood cell count; TSH: Thyroid-stimulating hormone; HbA1c: Hemoglobin A1c.

**Table 3:** Echocardiographic parameters of heart failure patients with and without ventricular tachycardia.

Variable	VT (−) (n = 86)	VT (+) (n = 57)	*p*-Value
LVEF (%)	38 ± 7.8	35.4 ± 9.2	0.074
LVEF ≤ 35%, n (%)	27 (31.4)	27 (48.2)	0.033
IVSD (mm)	11.9 ± 2.02	12 ± 2.3	0.911
LVPWD (mm)	10.6 ± 1.7	10.5 ± 1.5	0.861
Mitral E wave (cm/s)	76.2 ± 27.6	95.9 ± 37.5	<0.001
Mitral A wave (cm/s)	68.2 ± 26.1	78.5 ± 36.3	0.069
LVEDD (mm)	58.1 ± 8.7	58.5 ± 9.8	0.773
LVESD (mm)	46.7 ± 8.9	47.3 ± 10.5	0.772
Lateral e′ (cm/s)	8.2 ± 2.9	8.2 ± 2.7	0.844
Lateral a′ (cm/s)	7.3 ± 3.9	7.2 ± 3.1	0.972
Mitral deceleration time (ms)	164.3 ± 95.6	157.1 ± 74.9	0.635
LA maximum volume (mL)	24.7 ± 7.4	29.1 ± 9.2	0.003
LA minimum volume (mL)	19.2 ± 6.1	22.9 ± 8.1	0.003
LA pre-A volume (mL)	22.2 ± 6.5	26.1 ± 8.5	0.004
PAP (mmHg)	31.8 ± 15.8	31.9 ± 16.1	0.961
TAPSE (mm)	18.6 ± 4	18.1 ± 5.3	0.501
Epicardial fat thickness (mm)	0.28 ± 0.14	0.29 ± 0.15	0.678
E/A ratio	1.3 ± 0.76	1.48 ± 0.90	0.221
E/e′ ratio	10.5 ± 4.9	13 ± 7.22	0.013
MPI	0.61 ± 0.16	0.60 ± 0.20	0.836
Moderate/severe valvular disease, n (%)	39 (45.3)	27 (47.4)	0.473
Presystolic A wave, n (%)	11 (12.8)	6 (10.7)	0.463
MAC, n (%)	15 (17.4)	24 (43.6)	<0.001
Aortic sclerosis, n (%)	32 (37.2)	29 (52.7)	0.051
LAVI (mL/m^2^)	12.5 ± 3.6	14.4 ± 4.4	0.007

LVEF: Left ventricular ejection fraction; IVSD: Interventricular septal thickness; LVPWD: Left ventricular posterior wall thickness; LVEDD: Left ventricular end-diastolic diameter; LVESD: Left ventricular end-systolic diameter; LA: Left atrium; PAP: Pulmonary artery pressure; TAPSE: Tricuspid annular plane systolic excursion; MPI: Myocardial performance index; MAC: Mitral annular calcification; LAVI: Left atrial volume index.

**Table 4:** Independent predictors of ventricular tachycardia in heart failure patients.

Variable	Univariate OR (95% CI)	*p*-Value	Multivariable OR (95% CI)	*p*-Value
Age	1.030 (0.998–1.063)	0.068		
Sex	0.663 (0.265–1.657)	0.379		
NYHA III–IV	2.600 (0.989–6.838)	0.053		
Statin use	0.433 (0.218–0.865)	0.018		
Creatinine	2.870 (1.340–6.148)	0.007		
eGFR	0.982 (0.967–0.996)	0.015		
Albumin	0.330 (0.160–0.677)	0.003	0.372 (0.163–0.850)	0.019
Proteinuria	2.747 (1.294–5.832)	0.009		
WBC	1.233 (1.055–1.441)	0.009		
Neutrophils	1.298 (1.074–1.569)	0.007	1.279 (1.029–1.589)	0.027
Monocytes	7.061 (1.153–43.258)	0.035		
LVEF ≤ 35%	2.034 (1.016–4.074)	0.045		
Mitral E wave	1.019 (1.008–1.031)	0.001	1.019 (1.005–1.033)	0.009
E/e′ ratio	1.075 (1.013–1.141)	0.017		
MAC	3.665 (1.695–7.921)	<0.001	2.742 (1.130–6.653)	0.026
LAVI	1.131 (1.029–1.243)	0.011		

OR: Odds ratio; CI: Confidence interval; eGFR: Estimated glomerular filtration rate; WBC: White blood cell count; NYHA: New York Heart Association; MAC: Mitral annular calcification; LAVI: Left atrial volume index; LVEF: Left ventricular ejection fraction.

## Data Availability

The data supporting the findings of this study are not publicly available due to privacy and ethical restrictions but are available from the corresponding author upon reasonable request.
